# The climate crisis – can a community-led approach work?

**DOI:** 10.1177/17579139231180802

**Published:** 2023-06-29

**Authors:** BC O’Connor, M Hardman, LM Donkin, PA Cook

**Affiliations:** University of Salford, Allerton Building, Salford, M5 4WT, UK; University of Salford, Salford, UK; Bolton Council, Bolton, UK; University of Salford, Salford, UK

*Climate change is the biggest threat to human health: in this article, O’Connor et al. explore the question around whether a community approach could be beneficial to tackling climate change. It uncovers an unexplored area, namely sustainable community-level responses which deliver behaviour change for the climate emergency.*

**Figure:**
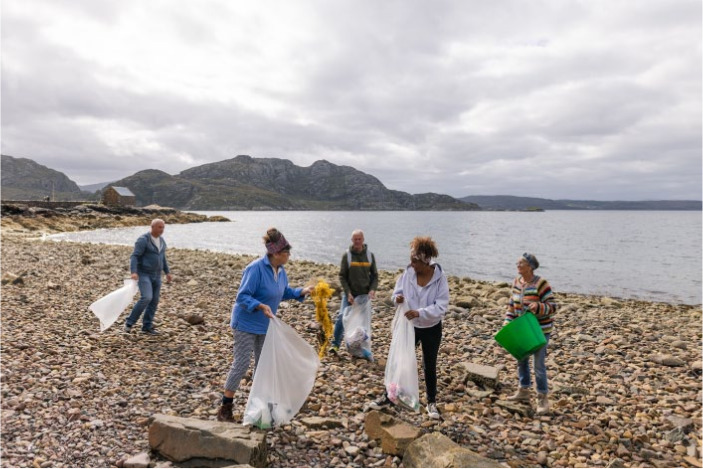


## Climate Change as a Threat to Human Health

Climate change is the biggest threat to human health.^1^ Over 300 Local authorities across the United Kingdom have declared climate emergencies,^2^ and there have been over 1500 declared by equivalent local authorities across the globe.^3^ Local authorities recognise the scale of the challenge and have set targets to reach ‘net zero’. Net zero, that is, not releasing more carbon dioxide than is captured, necessitates significantly reducing the amount of carbon dioxide emitted.^4^ Local authorities and the communities they serve are well placed to address this challenge both in terms of carbon emissions, as they “have powers or influence over roughly a third of emissions in their local areas” (p.3).^5^ and also through their connection and knowledge of their communities. This article asks the question: how can communities come together to make sustained change and accelerate progress towards net-zero?

## Justification for the Approach

The concept of communities coming together around a collective cause such as climate change is not new. As outlined in the Big Lottery Fund Report, communities working on climate change projects can lead to change. The challenge, as highlighted by the authors, is both the sustainability of the change and the impact towards reducing carbon emissions.^6^

The Local Government Association has several case studies highlighting community responses to the climate agenda.^7^ Such studies demonstrate meaningful engagement with the community around the climate; reach into groups not already engaged with the environmental agenda; and commitment to this approach. In Wiltshire, for example, two-thirds of the residents engaged in developing the priorities for the climate strategy were not already from environmental groups. However, the evidence of impact of this engagement is unclear, leading to the question: could the response have been strengthened by the community being involved in the delivery of the strategy? Other areas, such as Warwickshire, are investing in skills to support and build capacity in engagement, ensuring the voice of local people is woven into the development of the climate strategy.

A report from New Local around Climate Change and Community Action strongly advocates for a local response to the climate crisis.^8^ This puts the case forward that community-led action can be effective because of its ability to respond and mobilise quickly, adapt to climate impacts, and be an authentic approach because decisions are made locally. This power of community action has been demonstrated during the recent COVID-19 pandemic: in Bolton, the community was a key partner in helping to shape the response by providing assurance, delivering messages as a ‘trusted voice’, and developing community solutions.^9^ Climate change, like COVID-19, is an emergency but is arguably a greater challenge.^10^

The New Local report outlines examples where a community-led approach has galvanised action and led to changes that support low-carbon choices. The examples include: ‘Ambition Lawrence Wilson’, a community-driven project that has led to a community-owned solar farm; and the Cambridgeshire model, networking community groups and supporting residents to be ‘climate leaders’ through training and resources. Interestingly, this latter example has an element that is an evaluation of the carbon footprint of local organisations and businesses.

The above examples are promising; however, their effectiveness has not been evaluated. Further questions remain around whether a community approach can meaningfully contribute to a reduction in carbon emissions, whether this can be sustainable and whether it can build collective action and accelerate progress towards net zero. Moreover, it is not clear the extent of the reach and diversity of the communities they work with. Are they going beyond the ‘usual suspects’ and delivering sustainable change that would not have happened without the invention of council support or funding?

**Figure 1:**
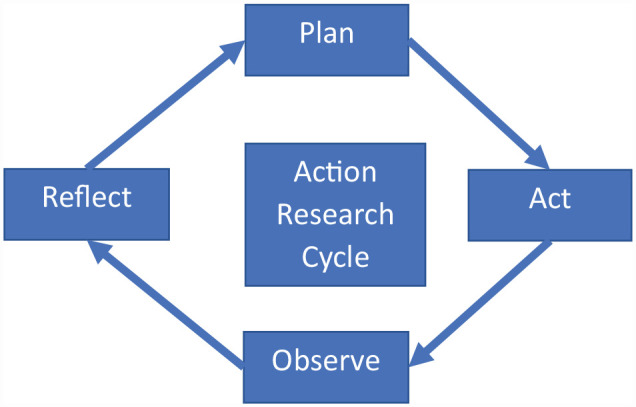
Stages in action research Source: Adapted from Costello,^12^ p. 7 © Costello, 2003, Action Research, Continuum, an imprint of Bloomsbury Publishing Plc.

Working ‘with’ and ‘for’ the community in the above examples has been a key ingredient to support community-led action. The gap in knowledge remains: how do we create that sustainable change, build momentum at a community-level, and evaluate whether and how it works? Action research might be a useful approach to explore how to bring diverse communities together to tackle climate change. This is because action research is collaborative and participatory following a continuous cycle of plan, act, observe, and reflect (see Figure 1). Action research creates ‘action’ and adds new knowledge ‘research’, addressing the question around which approach works or if it works. Moreover, the ethos of action research is that it is ‘with’ and ‘for’ the community rather than ‘at’ and ‘about’ the community.^11^

What evidence is there that an action research approach might work? In the realms of climate change, action research has mostly focused on climate adaptation and there is limited literature on using an action research approach for climate mitigation. Two studies from Australia and United States have used action research to design and implement policy change at a national and state level, respectively, in the field of climate mitigation.^13,14^ There remains an opportunity therefore to build on the methodology but with a focus on community-led action as a vehicle for delivering sustainable community behaviour change. We are currently trialling such an approach in Bolton, with co-researchers recruited from the community, using an action research approach.

## Conclusion

Community engagement and participatory approaches can support a community response. In some examples, these are a shared response between the council and the community and in others, they are truly community-led. However, many such projects have not been evaluated and the success factors are unclear. There is a gap in understanding what a sustainable response is; in other words, does it deliver behaviour change at a community-level? Finally, there remains the question, how do we build the momentum and collective community action seen for other emergencies, most notably COVID-19, for the climate emergency?
